# Reprogramming of Various Cell Types to a Beta-Like State by Pdx1, Ngn3 and MafA

**DOI:** 10.1371/journal.pone.0082424

**Published:** 2013-11-29

**Authors:** Ersin Akinci, Anannya Banga, Katie Tungatt, Joanna Segal, Daniel Eberhard, James R. Dutton, Jonathan M. W. Slack

**Affiliations:** 1 Stem Cell Institute, University of Minnesota, Minneapolis, Minnesota, United States of America; 2 Department of Biology and Biochemistry, University of Bath, Bath, United Kingdom; University of British Columbia, Canada

## Abstract

The three transcription factors, PDX1, NGN3 and MAFA, are very important in pancreatic development. Overexpression of these three factors can reprogram both pancreatic exocrine cells and SOX9-positive cells of the liver into cells resembling pancreatic beta cells. In this study we investigate whether other cell types can be reprogrammed. Eight cell types are compared and the results are consistent with the idea that reprogramming occurs to a greater degree for developmentally related cells (pancreas, liver) than for other types, such as fibroblasts. Using a line of mouse hepatocyte-derived cells we screened 13 compounds for the ability to increase the yield of reprogrammed cells. Three are active and when used in combination they can increase the yield of insulin-immunopositive cells by a factor of six. These results should contribute to the eventual ability to develop a new cure for diabetes based on the ability to reprogram other cells in the body to a beta cell phenotype.

## Introduction

Diabetes mellitus is a life threatening metabolic disease the prevalence of which is increasing worldwide [1]. It is characterized by hyperglycemia due to an absolute lack of insulin from pancreatic beta cells (Type 1 diabetes) or a relative lack (Type 2 diabetes). Complications of diabetes such as cardiovascular diseases, retinopathy, neuropathy, nephropathy, and peripheral circulatory diseases depend on imperfect regulation of blood sugar and can be lethal if they are not treated. Despite its good effectiveness, the therapy provided by insulin injections cannot reproduce the normal insulin secretion pattern as efficiently as beta cells. Beta cell transplantation is effective to some degree but the shortage of cadaveric pancreases is a major limitation, and immune suppression is necessary, which causes side effects and toxicity to the graft [2]. These limitations could potentially be overcome by reprogramming of other cells within the body of the patient into insulin-expressing, glucose-sensitive beta-like cells [3]. Production of new beta cells from highly regenerative organs such as liver or from organs in which alteration of some cells does not affect the overall function, such as the exocrine pancreas, would also solve the problem of the shortage of cells for transplantation. Based on this possibility, many studies regarding beta cell reprogramming have been performed in liver cells both *in vivo* and *in vitro*. Recently, overexpression of *Pdx1*, *Ngn3* and *MafA* in the exocrine pancreas of mouse was shown to produce insulin-positive cells which were capable of rescuing RAG1-/- mice made diabetic by treatment with streptozotocin [4]. We have followed up this study using a single adenovector encoding all three factors. Our study on the rat AR42j-B13 cell line, which has a pancreatic exocrine phenotype, indicated that the transformation is reproducible and stable, but does not confer all the beta cell properties, especially the critical property of glucose-sensitivity [5]. Recently we showed that the same gene combination was able to induce the formation of insulin-secreting, glucose-sensitive ductal structures in the livers of immunodeficient mice, and the cell of origin was identified as a SOX9-positive population, either small bile ducts or perhaps bipotential progenitor cells located in the periportal regions of the liver [6]. In this case the reprogrammed cells were glucose-sensitive. The combination *Pdx1*, *Ngn3* and *MafA* (here abbreviated to PNM) represents a logical gene set for stimulating pancreatic endocrine development. In the normal embryo *Pdx1* is required for pancreatic bud outgrowth, *Ngn3* for endocrine precursor cell formation, and *MafA* (and *Pdx1* again), for beta-cell maturation [7].

In the current study we have extended our understanding of PNM effects in two respects. First we have looked at the reprogramming competence of various different cell types. The cells we used were mouse hepatocyte-derived small cells (ASH cells), mouse primary hepatocytes, mouse embryonic fibroblasts (MEF) and mouse adult (tail tip) fibroblasts, rat primary hepatocytes, rat pancreatic exocrine cells (AR42J-B13), rat adult fibroblasts (CRL-1213) and rat multipotent adult progenitor cells (MAPC). The results are consistent with the idea that reprogramming occurs to a greater degree for developmentally related cells (pancreas, liver) than for fibroblasts.

Secondly, we have investigated the effect of a panel of small molecules which are candidates for improving reprogramming efficiency, together with the three transcription factors. For this we used the mouse hepatocyte-derived small cells, which normally show a reproducible but low percentage of transformation. We found three substances: DAPT, an antagonist of Notch signaling, NECA, an adenosine agonist, and BIX-01294, an inhibitor of histone deacetylases, each of which individually increases reprogramming to some degree and together do so by a factor of 6. We expect these substances to be useful for reprogramming procedures in the future.

## Materials and Methods

### Cell Culture and Viral Transfection

Mouse hepatocyte-derived small cells (ASH cells) were derived from primary mouse hepatocytes of a mouse of *AlbCre;R26R* genotype, and are known by the abbreviation ASH (="*AlbCre* small hepatocytes”). The hepatocytes were isolated at the University of Bath using the two step collagenase perfusion method [8]. This is a non-survival procedure conducted under anesthesia. The work was conducted under Home Office Project Licence PPL30/2004 "Transdifferentiation of Rodent Tissues" granted to JMWS. The cells were maintained in low-glucose Dulbecco`s Modified Eagles medium (DMEM; Gibco) supplied with 10% (v/v) fetal bovine serum (FBS; Hyclone) and 1x antibiotic-antimycotic solution (anti-anti; Gibco). Because they are derived from albumin-positive precursors they express the β-geo marker, although this was not used in the present work. Their small size and rapid growth rate may indicate a "progenitor-like" status, although their exact *in vivo* counterpart is not known.

Mouse embryonic fibroblasts (MEF) were isolated in the laboratory from prenatal mouse embryos. The pregnant females were sacrificed by the approved method of CO_2_ overdose under University of Minnesota IACUC Protocol 1002A78295 "Directed Transdifferentiation of Rodent Tissues" granted to JMWS. Cells were maintained in high-glucose DMEM supplied with 10% (v/v) FBS, 2mM L-glutamine (Gibco) and 1x anti-anti solution.

Mouse adult fibroblasts were obtained in the laboratory by culturing a piece of tail tip from an adult mouse *in vitro*. The mouse was sacrificed by the approved method of CO_2_ overdose under University of Minnesota IACUC Protocol 1002A78295 "Directed Transdifferentiation of Rodent Tissues" granted to JMWS. The fibroblasts were maintained in high-glucose DMEM supplied with 10% (v/v) FBS, 2mM L-glutamine and1x anti-anti solution.

Rat and mouse primary hepatocytes were isolated from adult male rats and mice by the two-step collagenase perfusion method [8] under University of Minnesota IACUC Protocol 1002A78295 "Directed Transdifferentiation of Rodent Tissues" granted to JMWS. After the isolation they were cultured in Williams' E medium (Gibco) supplied with 10% (v/v) FBS, 2mM L-glutamine, 1x anti-anti and gentamycin solution (Gibco) for overnight for attachment to plate. After the cells attached to the plate they were maintained in low-glucose DMEM supplied with 10% (v/v) FBS, 2mM L-glutamine and 1x anti-anti solution.

Rat pancreatic exocrine-like cells (AR42J-B13), which were originally derived from a chemically induced pancreatic tumor [9], were obtained from Dr David Tosh (University of Bath, UK) and maintained in low-glucose DMEM supplied with 10% (v/v) FBS, 2mM L-glutamine and 1x anti-anti solution.

Rat adult fibroblast cells (CRL-1213) were purchased from ATCC. They were maintained in high-glucose DMEM supplied with 10% (v/v) FBS, 2mM L-glutamine and 1x anti-anti solution.

Rat multipotent adult progenitor cells (MAPC) were obtained from Dr Wei-Shou Hu`s Laboratory (University of Minnesota, MN). These cells originate from adult bone marrow but have some properties of embryonic stem cells [10]. They were maintained in low-glucose DMEM and MCDB media (Sigma) supplied with 2% (v/v) FBS, 1x anti-anti solution, 1x ITS (Insulin/Transferrin/Selenium; Sigma), 1mg/ml LA-BSA (Linoleic acid-bovine serum albumin; Sigma), 1x L-ascorbic acid, 0.5µM dexamethasone, 10ng/ml PDGF (Platelet-derived growth factor; Sigma), 10ng/ml EGF (Epidermal growth factor; Millipore), 0.01x LIF (Leukemia inhibitory factor; Millipore) and 0.1x β-mercaptoethanol (Gibco). 

An adenovector (*Ad-PNM*) carrying mouse *Pdx1*, *Ngn3* and *MafA* in a single transcription unit, separated by 2A sequences, was prepared as described previously [5]. Preliminary trials showed that a multiplicity of infection (MOI) of 25 gave maximum insulin-positive cells with minimum toxicity so this dose was adopted as the standard for the study. Next day the virus-containing medium was replaced. Three days after virus transduction, total RNA was isolated for reverse transcription-polymerase chain reaction (RT-PCR) and the cells were also fixed for immunostaining to see how they had responded. The *Ad-PNM* infection efficiency was calculated for each cell type to ensure it was sufficient to give reliable results. The efficiency was obtained by counting all the cells in three fields of vision and taking an average percentage of the cell population showing nuclear PDX1 immunostaining. 

### Reverse Transcription-Polymerase Chain Reaction (RT-PCR)

 Total RNA from the cells was isolated using the RNeasy Mini Kit (Qiagen). RNA samples were then treated with DNase enzyme (Promega) to remove possible genomic DNA contamination. cDNAs were then synthesized by reverse transcription from 2μg total RNA using SuperScript III Reverse Transcriptase, oligo(dT)_20_ and dNTP (10mM) (Invitrogen). The gene expression pattern between the cells with and without *Ad-PNM* was then compared by performing PCR. After an initial denaturation at 94°C for 3 min, reaction mixtures were subjected to 20-35 cycles of amplification using the following conditions: 94°C 30 s denaturation, 60°C 30 s annealing, 72°C 1 min extension, 72°C 5 min final extension. PCR products were then run on 1% (w/v) agarose gel (Invitrogen), in TAE (Tris-Acetic Acid-EDTA) buffer (Bio-Rad Laboratories) including ethidium bromide, by electrophoresis at 100V. Gels were visualized with a Bio-Rad Gel Documentation System model 2000. Primer sequences are available on request. For these experiments the negative control was water. The positive control was whole pancreas RNA for markers expressed in pancreas. For other markers it was as follows: whole liver RNA for *Alb*, embryonic liver bud RNA for *Afp*, mouse and rat adult fibroblast RNA for *Vim*, rat RNA for *Oct4*, donated by the lab of Dr Hu (UofM).

### Quantitative Reverse Transcription–Polymerase Chain Reaction (qRT-PCR)

Total RNA isolation and cDNA synthesis were performed as described above. The gene expression pattern between the cells with and without *Ad-PNM* was then compared by performing qRT–PCR. After an initial denaturation at 95°C for 30 s, reaction mixtures were subjected to 40 cycles of amplification using the following conditions: 95°C for 5 s denaturation, and 60°C for 10 s annealing and extension. Primer sequences are available on request. The expression levels of the genes of interest were expressed relative to transcripts of the gene for the ubiquitous enzyme glyceraldehyde-3-phosphate dehydrogenase (GAPDH), which was normalized to zero.

### Immunostaining

The cells were fixed with 4% (w/v) paraformaldehyde (Sigma) in PBS (phosphate buffered saline; Sigma) for 20 min. After fixation cells were washed three times with 0.1% (v/v) Tween 20 (Bio-Rad) in PBS (PBS-T) for 5 min in each wash. Cells were then permeabilized with 0.2% (v/v) Triton X-100 (Sigma) in PBS for 15 min. After 1 hr blocking with PBS-T including 1% (w/v) BSA (Bovine serum albumin; Sigma), cells were incubated overnight with primary antibody at 4°C and then 1 hr with secondary antibody at room temperature. Primary antibodies used were as follows: guinea pig anti-insulin (1:200, Sigma); rabbit anti-PDX1 (1:2000, Upstate); rabbit anti-C-peptide (1:100, Cell Signaling), rabbit anti-NGN3 (1:100, Santa Cruz Biotechnology); rabbit anti-MAFA (1:100, Santa Cruz Biotechnology). Secondary antibodies used were as follows: alexa fluor 480 goat anti-rabbit IgG (1:500, Invitrogen) and alexa fluor 594 goat anti-guinea pig IgG (1:500, Invitrogen). Images were taken using a Leica DMI6000 B inverted microscope. 

### Administration of Small Molecules with *Ad-PNM*

10^5^ mouse hepatocyte-derived small cells (ASH cells) were plated into each well of 6-well plates (Day 0). The cells were allowed to attach overnight. The next day each substance was individually given to the cells in different wells at the final concentrations mentioned below (Day 1). After two days incubation, the number of the cells per well was calculated and 15 MOI *Ad-PNM* was given without removing the substances from the medium (Day 3). The next day, both *Ad-PNM* and the substance-containing medium was removed (Day 4). The cells were maintained in the culture for two more days before analysis. 

The substances were: trichostatin A (TSA), valproic acid (VPA), suberoylanilide hydroxamic acid (SAHA), BIX-01294, 5-azacytosine (5-AzaC), pirinixic acid (WY-14643), *N*-[*N*-(3,5-difluorophenacetyl)-l-alanyl]-*S*-phenylglycine *t*-butyl ester (DAPT), mycophenolic acid (MPA), 5'-*N*-ethylcarboxamidoadenosine (NECA), nicotinamide, retinoic acid (RA), disulfiram (tetraethylthiuram disulphide) and diethylaminobenzaldehyde (DEAB).

Concentrations used were as follows: 1 mM for TSA (Sigma, diluted in dH_2_O), 1 mM for VPA (Sigma, diluted in dH_2_O), 5 μM for SAHA (Sigma, diluted in DMSO), 2 μM for BIX (Tocris, diluted in dH_2_O), 5 μM for 5-AzaC (Sigma, diluted in DMSO), 75 μM for WY-14643 (Cayman, diluted in DMSO), 10 μM for DAPT (Sigma, diluted in DMSO), 1 μM for MPA (Sigma, diluted in DMSO), 10 μM for NECA (Tocris, diluted in DMSO), 5 mM for nicotinamide (Sigma, diluted in dH_2_O), 100 nm for disulfiram (Sigma, diluted in DMSO), 10 μM for DEAB (Aldrich, diluted in DMSO), 10 μM for RA (Sigma, diluted in DMSO).

To see if the small molecules increase the reprogramming efficiency of *Ad-PNM*, we counted the number of insulin-expressing cells. For this purpose, the cells were fixed and immunostained for insulin and PDX1 (Day 6). The number of insulin-positive cells as a fraction of PDX1-positive cells was calculated by counting ten randomly chosen different areas in each well. Three different wells were counted for each experimental group and two different experiments were set up at different times. One way ANOVA tests were used to make statistical comparisons.

### ELISA for glucose-stimulated insulin release

5x10^5^ cells were plated in each well of a six-well plate. The medium was removed and cells were incubated in Krebs Ringer Buffer (KRB) without glucose for two hours at 37°C. They were then exposed to 2.8 mM or 20 mM glucose in KRB for 1 hour at 37°C. The medium was then removed and kept at -20°C for measurement of secreted insulin using an Ultrasensitive Insulin ELISA kit (Mercodia) according to the manufacturer's manual. One way ANOVA with Tukey posthoc tests and additional t tests were used to make statistical comparisons.

For measurement of insulin content, the cells were detached with trypsin and resuspended in KRB. A sample was used for a total protein measurement with the Pierce BCA Protein Assay kit (Thermo Scientific). After homogenization using an insulin syringe, the cells, with protease inhibitors, were rotated overnight at 4°C and centrifuged (12000 rpm, 5 min at 4°C). Then the supernatant was collected and the insulin concentration was measured as above. 

## Results

**Figure 1:**
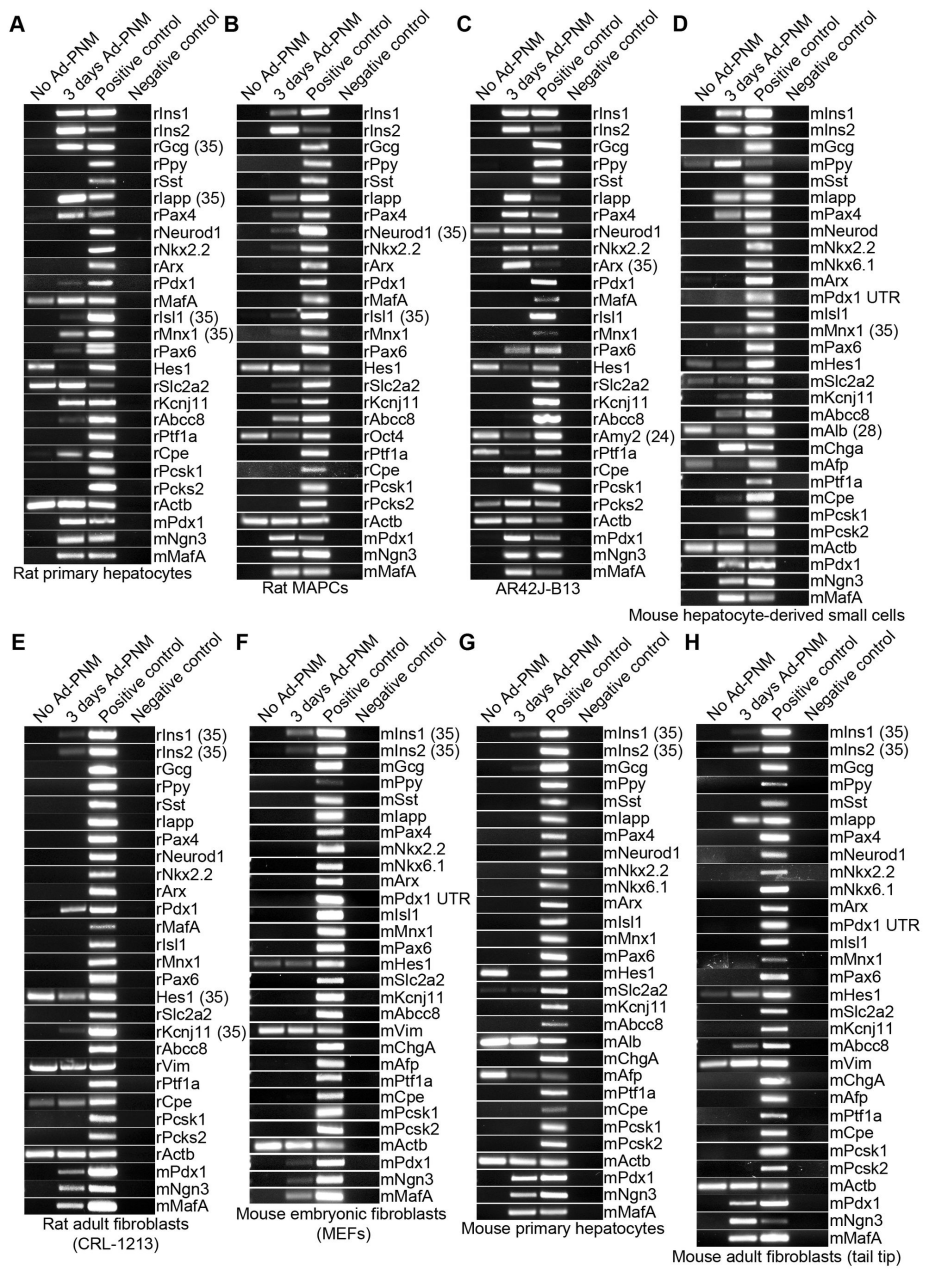
Expression of various beta cell genes provoked by transduction with *Ad-PNM*. A-H. Qualitative RT-PCRs showing the effect of *Ad-PNM* on the expression of a panel of beta cell genes. 30 cycles were used unless otherwise shown. The misaligned bands in the first two lanes of mouse fibroblast *Mnx1* are primer dimers, not RNA.

**Figure 2:**
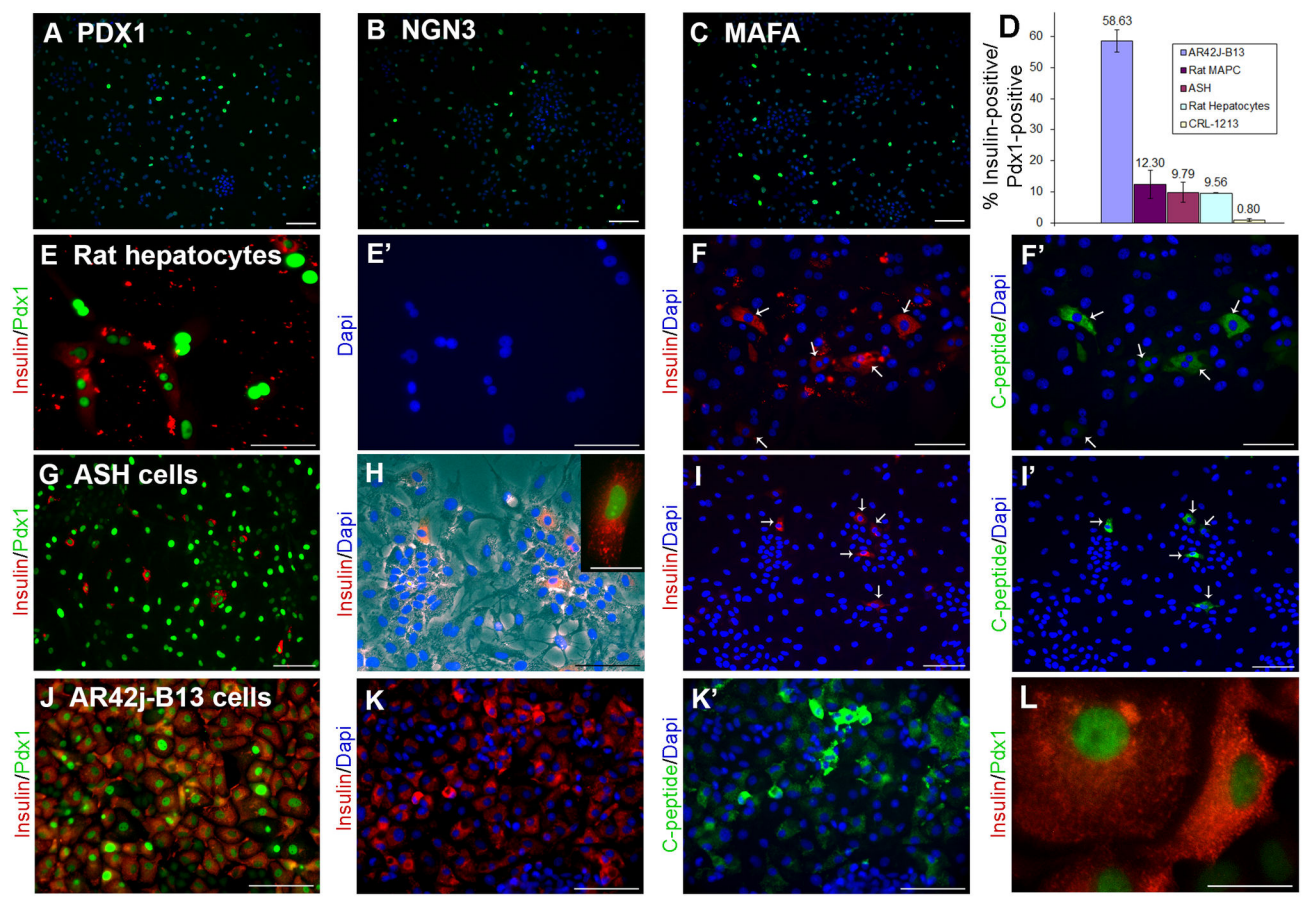
Insulin-positive cells generated from different cell types. A,B,C Expression of PDX1, NGN3, MAFA in ASH cells. The proteins are shown in green, DAPI in blue. D. Histogram of the percentage PDX1-positive cells which are insulin-positive in different cell types. E,E’ Rat hepatocytes, some PDX1-positive cells are insulin-positive. F,F’ Rat hepatocytes, insulin-positive cells are also C-peptide-positive. G Mouse ASH cells, some PDX1-positive cells are insulin-positive. H. Mouse ASH cells, showing cytoplasmic location of insulin. Inset, high magnification showing insulin-containing granules. I,I’ Mouse ASH cells showing co-expression of C-peptide with insulin. J Rat AR42j-B13 cells showing a high proportion of insulin-positive cells. K,K’ Rat AR42j-B13 cells showing co-expression of C-peptide with insulin. L. Rat AR42j-B13 showing insulin-containing granules. Scale bars 100µm, except H inset and L which are 25 µm. Blue color is DAPI throughout.

A total of eight different cell types from rat and mouse were treated with *Ad-PNM*, our adenovector which contains *Pdx1, Ngn3* and *MafA* coding regions as a single transcription unit, separated by 2A sequences. The four rat cells were: pancreatic exocrine cells (AR42J-B13), primary hepatocytes, adult fibroblasts (CRL-1213) and multipotent adult progenitor cells (MAPC). The four mouse cells were hepatocyte-derived small cells (ASH), primary hepatocytes, tail tip fibroblasts and embryonic fibroblasts (MEF). Three days after viral transduction the cells were processed for analysis. Three days was selected as a suitable time point for several reasons. Previous studies with B13 cells had indicated little change in response after three days [5]. Some of the cell types, such as ASH, are highly proliferative and this means that a longer time period enables the parent cells to overgrow the transformed ones. Conversely, some of the primary cells, particularly the primary hepatocytes, show significant cell death beyond three days culture. For cell characterization, total RNA was isolated for RT-PCR for a range of beta cell markers (Figure **1**), and cells were fixed for immunostaining (Figure **2**). The cells were co-stained for insulin expression, for the *Ad-PNM* input gene products, and for C-peptide. 

The level of viral transduction for this set of experiments was as follows, given as % PDX1-positive ± standard error: AR42j-B13 96.5±0.19; rat primary hepatocytes 98.7±0.29; rat fibroblasts 92.6±0.87; rat MAPC 43.8±2.09; mouse ASH 80.2±3.92; mouse primary hepatocytes 98.9±0.69, mouse tail tip fibroblasts 81.7±3.57; mouse embryonic fibroblasts 66.1±7.38. Cells were also stained for NGN3 and MAFA, which were found at similar levels to PDX1 (Figure **2A-C**). 

In all eight cell types used in this study, three days after *Ad-PNM* transduction, *insulin* gene expression was detected by RT-PCR (rodents have two *insulin* genes, *Ins1* and *Ins2*) (Figure **1**). In addition to *insulin*, the most responsive four cell types (rat primary hepatocytes, rat MAPCs, AR42J-B13 cells and mouse small hepatocytes) also upregulated some other important beta cell markers (Figure **1A-D**). Rat primary hepatocytes started to express the beta cell transcription factor genes *Pax4*, *Isl1* and *Mnx1*, the beta cell membrane protein genes *Abcc8*, *Kcnj11* (=*Sur2, Kir6.2*, encoding components of the ATP-sensitive K channel, important in insulin secretion), and *Slc2a2* (*=Glut2*, encoding the β-cell glucose transporter), the endogenous counterpart of *Pdx1*, and *Cpe* (*Carboxypeptidase E*, encoding an enzyme important for proinsulin processing). They also upregulated the gene for Iapp (islet amyloid polypeptide), and the alpha cell hormone gene *glucagon* (*Gcg*). Rat MAPCs started to express the beta cell transcription factor genes *Pax4*, *Neurod1*, *Nkx2.2*, *Isl1*, *Mnx1*; the beta cell membrane protein genes *Slc2a2*, *Kcnj11* and *Abcc8*. AR42J-B13 cells started to express the beta cell transcription factor genes *Pax4*, *Neurod1*, *Nkx2.2*; the beta cell membrane protein gene *Abcc8* as well as *Cpe* and *Pcsk2*. Mouse ASH cells started to express the beta cell transcription factor genes *Pax4* and *Mnx1*, the beta cell membrane protein genes *Slc2a2*, *Kcnj11* and *Abcc8*; as well as *Cpe* and *Pcsk2* (*Proprotein convertase subtilisin/kexin type 2*).They also expressed the PP-cell hormone gene *Ppy* (*Pancreatic polypeptide*). All these cell types also upregulated *Iapp*. In addition to upregulating some of the various beta cell genes, the various cell types also downregulated the expression of some of their original specifically expressed genes, including *Pou5f1* (*= Oct4*) in rat MAPCs, *Amy2* (*pancreatic amylase*) in AR42J-B13 cells, and *Alb* (*albumin*) in mouse hepatocyte-derived small cells. The other four cell types: rat adult fibroblasts (CRL-1213), mouse embryonic fibroblasts (MEF), mouse primary hepatocytes and mouse adult fibroblasts (tail tip) showed little or no response of the beta cell genes apart from the *insulin* genes (Figure **1E-H**).

Although *insulin* gene expression was detected for all the cell types by RT-PCR, insulin immunostaining was only detected in five cell types, which are rat primary hepatocytes, mouse hepatocyte-derived small cells (ASH), AR42J-B13 cells, rat MAPCs and CRL-1213, and only reliably for the first three (Figure **2D-L**). In addition to insulin, the presence of C-peptide, which is a product formed during the cleavage of proinsulin to insulin, and its co-localization with insulin in the same cells, were also shown for rat primary hepatocytes, mouse hepatocyte-derived small cells and AR42J-B13 cells (Figure **2F'** **,I**',**K**’). 

The qualitative RT-PCR data are summarized in Tables **1** and **2**, and counts of insulin-positive cells as a fraction of transduced cells (Pdx1-positive) are given in Figure **2D**. There was a highly statistically significant difference between groups as determined by one-way ANOVA (F(4,10) = 59.856, p < 0.001). To confirm where the differences occurred between groups we performed a Tukey post-hoc test. This test is appropriate for data showing homogeneity of variances between groups. It showed a significant difference (p<0.01) between B13 and each of the other cell types, but a non-significant difference (p>0.1) between each of the other cell types with each other. 

**Table 1:** Upregulation of gene expression by *Ad-PNM* in the four rat cell types.

**Pancreatic mRNA**	**AR42j-B13**	**Hepatocytes**	**CRL-1213**	**MAPC**
*rInsulin1*	++++	++++	+	++
*rInsulin2*	++++	++++	+	++++
*rGlucagon*	-	+++	-	-
*rPanc.Polypeptide*	-	-	-	-
*rSomatostatin*	-	-	-	-
*rIAPP*	+++	++++	-	++
*rPax4*	+++	+++	-	++
*rNeuroD*	C	-	-	+
*rNkx2.2*	+	-	-	+
*rArx*	+++	-	-	+
*rPdx1*	-	+	+	-
*rMafA*	-	C	-	-
*rIsl1*	-	+	-	+
*rMnx1*	-	++	-	+
*rPax6*	+	+	-	-
*rHes1*	C	C	C	C
*rGlut2(Slc2a21)*	-	C	-	+
*rKir6.2((Kcnj11)*	+	+++	+	+
*rSur1(Abcc8)*	-	+	-	++
*rPtf1a*	C	-	-	-
*rCarboxypeptidaseE*	+++	++	C	-
*rPcsk1*	-	-	-	-
*rPcsk2*	C	-	-	-

++++,+++,++,+ indicate approximate expression levels, - means not detected, C means present in untreated cells of this type.

**Table 2:** Upregulation of genes by Ad-PNM in the four mouse cell types.

**Pancreatic mRNA**	**ASH**	**Hepatocytes**	**Tail tip fibroblasts**	**MEF**
*mInsulin1*	++++	+	+	++
*mInsulin2*	++++	-	++	+
*mGlucagon*	-	+	+	-
*mPanc.Polypeptide*	++	-	-	-
*mSomatostatin*	-	-	-	-
*mIAPP*	+++	-	+++	-
*mPax4*	++	-	-	-
*mNeuroD*	-	-	-	ND
*mNkx2.2*	-	-	+	-
*mNkx6.1*	-	-	-	-
*mArx*	C	-	-	-
*mPdx1 UTR*	-	-	-	-
*mIsl1*	-	-	-	-
*mMnx1*	+	-	-	-
*mPax6*	-	-	-	-
*mHes1*	C	C	C	C
*mGlut2 (Slc2a21)*	C	C	-	-
*mKir6.2 (Kcnj11)*	+	-	-	-
*mSur1 (Abcc8)*	++	-	++	-
*mChromogranin*	++++	-	-	-
*mAfp*	C	C	-	-
*mPTF1a*	-	-	-	-
*mCarboxypeptidaseE*	+	-	+	-
*mPcsk1*	-	-	-	-
*mPcsk2*	+	-	-	-

++++,+++,++,+ indicate approximate expression levels, - means not detected, C means present in untreated cells of this type, ND not determined.

**Figure 3:**
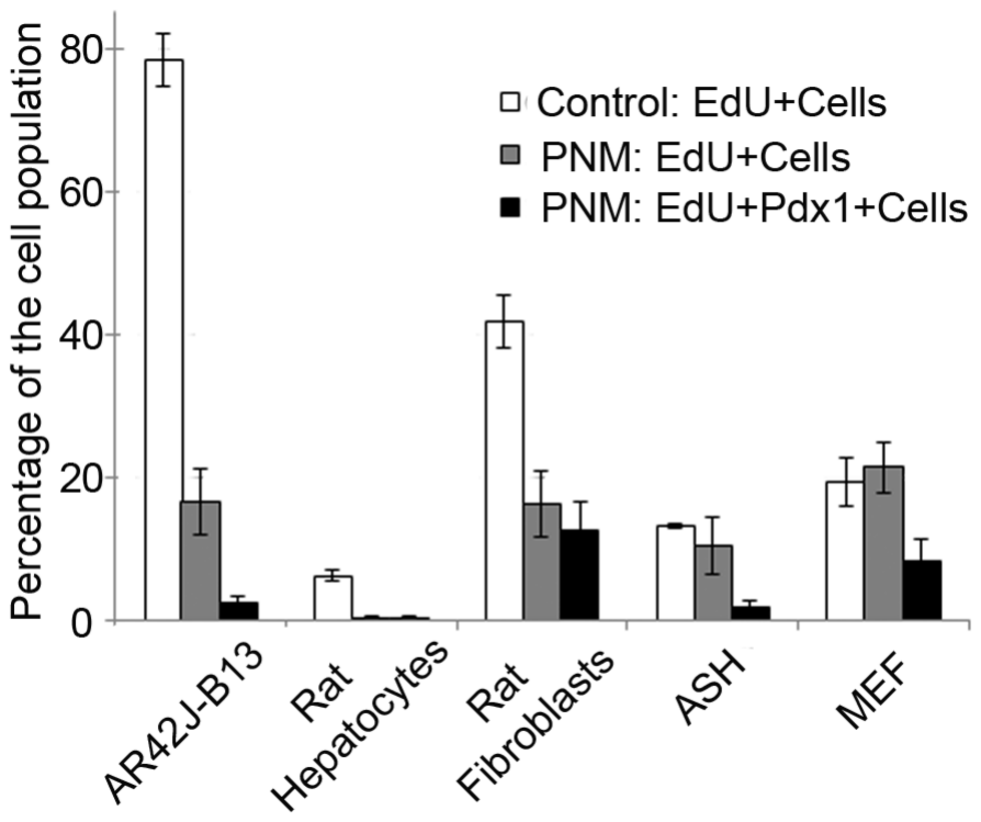
EdU labeling index in five cell types. Those which generated insulin-immunopositive cells showed a reduction in EdU label. Errors are standard errors, n=3.

We also examined the effect of the *Ad-PNM* transduction on the division rates of the cells. Ethynyl deoxyuridine (EdU) was administered three days after *Ad-PNM* and the cells fixed 18 hours later. The percentage of S-phase cells in the control populations was compared to the percentage of S-phase cells among the virus-transduced population (calculated as the percentage positive for (PDX1+EdU)/PDX1). This indicated a considerable drop in the proportion of dividing cells for the AR42j-B13 cells and hepatocytes, but not for the fibroblasts, maybe because the degree of reprogramming for the fibroblasts was very limited (Figure **3**). For PDX1-positive cells there was a significant drop in EdU index compared to the overall score for *Ad-PNM* transduced plates (t-tests: p<0.05 for AR42j-B13, ASH and MEF). If only insulin-positive cells are scored for EdU labeling, then the percentages are very low (AR42j-B13: 2.89±1.52; rat hepatocytes: 0, ASH cells 0; other cell types showed insufficient insulin-positive cells for scoring) suggesting that reprogramming to the beta-like state is not compatible with continued cell division. Because of this lack of further division, it is not possible to maintain the insulin-positive cells long term. However they have been maintained in viable but non-dividing form for up to 4 weeks and so we believe that the reprogramming is stable and not just a transient gene expression induced by the vector-encoded factors. The phenotype of the insulin-positive cells derived from the pancreatic exocrine (AR42j-B13) cells has been characterized in detail and published separately [5]. That study showed that it represents a partial transformation toward a beta-cell phenotype, not a complete one.

### Enhancement of reprogramming by small molecules

Although a high proportion of *Ad-PNM* transduced AR42j-B13 cells will become immunopositive for insulin, for the rat hepatocytes and ASH cells the percentage of insulin-positive cells produced by *Ad-PNM* is low. We were interested to find whether adjuvant treatments with small molecule-type substances could increase the yield. The mouse small hepatocytes (ASH cells) were chosen to test the effects of 13 different compounds. ASH cells have a number of practical advantages over primary hepatocytes; they are rapidly expandable, homogeneous, have a stable phenotype, and are smaller and less fragile than hepatocytes. Furthermore a consistent percentage of cells became insulin-positive following *Ad-PNM* treatment. In this series of experiments, 2% of the ASH cells that were positive for PDX1 were also positive for insulin. This is a low percentage which easily allows increases to be detected. We confirmed that all the insulin-positive cells are PDX1-positive and no insulin-positive cells are ever found in untransduced cultures.

We chose thirteen different small molecule agents that were either shown or thought to favor beta cell formation, replication and/or survival in other systems. We classified them into three groups: a) chromatin modification agents: trichostatin A (TSA), valproic acid (VPA), suberoylanilide hydroxamic acid (SAHA), BIX-01294 (BIX for short, a diazepin-quinazolin-amine derivative) and 5-azacytosine (5-AzaC); b) signaling/metabolism agents: pirinixic acid (WY-14643) (an agonist of PPARα and γ), *N*-[*N*-(3,5-difluorophenacetyl)-l-alanyl]-*S*-phenylglycine *t*-butyl ester (DAPT) (an inhibitor of Notch), mycophenolic acid (MPA) (an inhibitor of IMP dehydrogenase which is used as an immunosuppressant), 5'-*N*-ethylcarboxamidoadenosine (NECA) (an agonist of adenosine) and nicotinamide (precursor of NAD/NADP, and inhibitor of PARP-1); c) retinoic acid (RA) and the inhibitors of its synthesis disulfiram (tetraethylthiuram disulphide) and diethylaminobenzaldehyde (DEAB).

**Figure 4:**
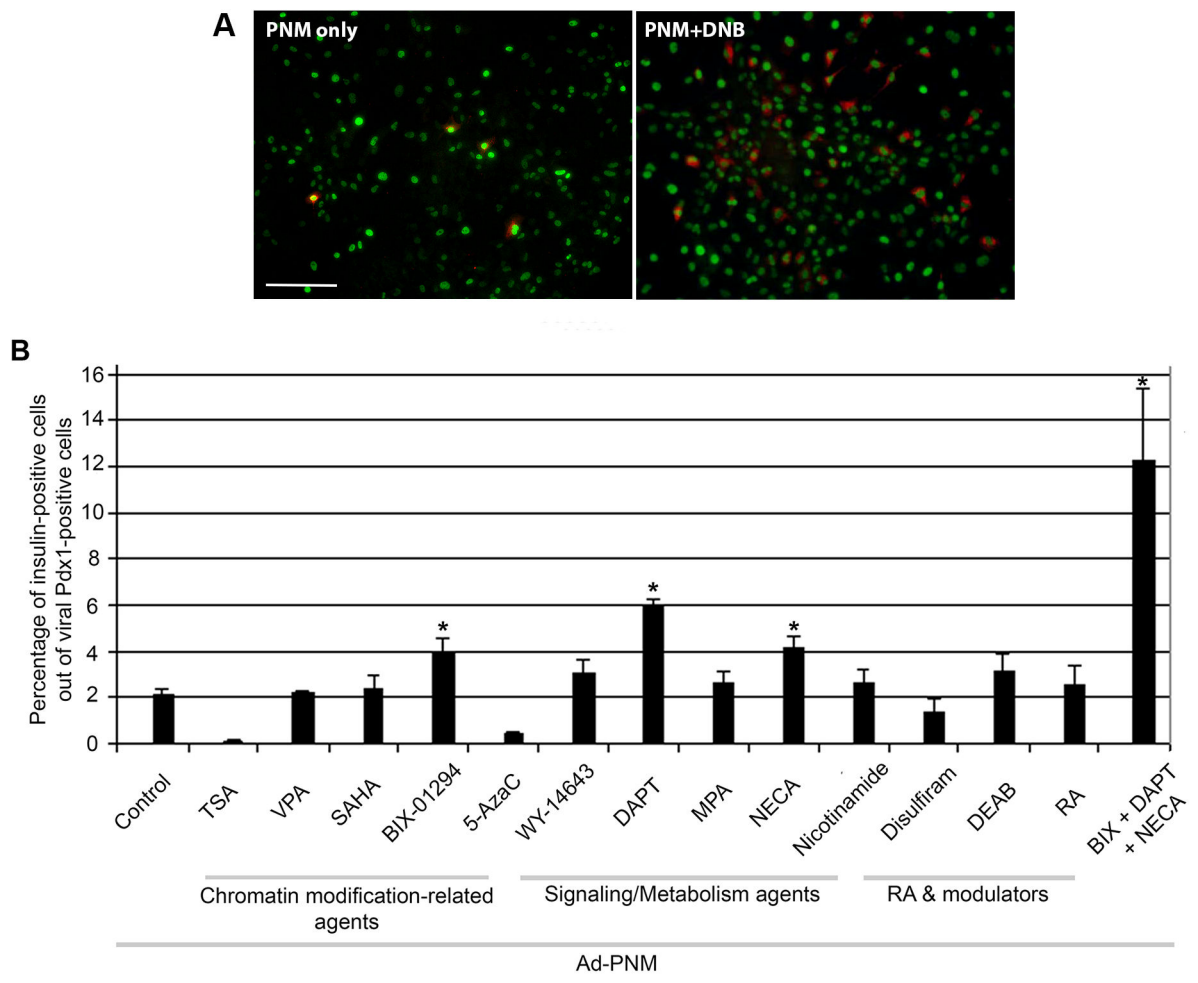
Effects of the small molecule combination. A. ASH cells transduced with *Ad-PNM*, without and with the DNB combination. PDX1 is immunostained green and insulin red. Scale bar 100µm. B. Percentage of PDX1-positive cells that are also insulin-positive. Comparisons are by one way ANOVA with Games-Howell post hoc test, * indicates significant enhancement. Errors are standard errors, n=6.

The concentrations used were those that were just below the level at which detectable cell death was produced over a two day culture. Each of the substances was added to the cells two days before *Ad-PNM* addition. Three days after viral transduction the percentage of insulin-positive cells was calculated, as a fraction of PDX1-positive cells. Results were analyzed by one way analysis of variance followed by the Games-Howell post hoc test (appropriate here because the variances are not homogeneous). There was a highly significant difference between groups as determined by one-way ANOVA (F(14,54) = 22.477, p < 0.001). From the 13 substances screened, three increased to a significant degree the number of insulin-positive cells as a fraction of the PDX1-positive cells (Figure **4**). These were DAPT (p<0.001), NECA (p=0.007) and BIX (p=0.049). They yielded respectively ~6% (3 fold increase), ~4% (2 fold increase) and ~4% (2 fold increase). TSA and 5-azaC reduced the insulin-positive cells but also had a noticeable toxic effect, even at low concentrations, and completely stopped the cells from dividing. The other substances had no significant effect on the yield of insulin-positive cells.

DAPT, NECA and BIX all exert their biochemical effects by different mechanisms and therefore we felt that in combination they should have an additive effect on the number of insulin-positive cells. When ASH cells were exposed to all three of these substances at the same time, there was a significant amount of cell death. After some experimentation, the concentration of NECA and BIX was reduced to half the original, with the concentration of DAPT remaining as previously. So the three substance combination DNB consisted of DAPT:10µM, NECA:5µM, BIX:1µM. Cells in DNB stayed in good health and, as shown in Figure **4**, showed a significantly (p=0.006) better response to *Ad-PNM* than to any of the substances alone. The proportion of insulin-positive cells under these conditions was about 12 per cent. 

**Figure 5:**
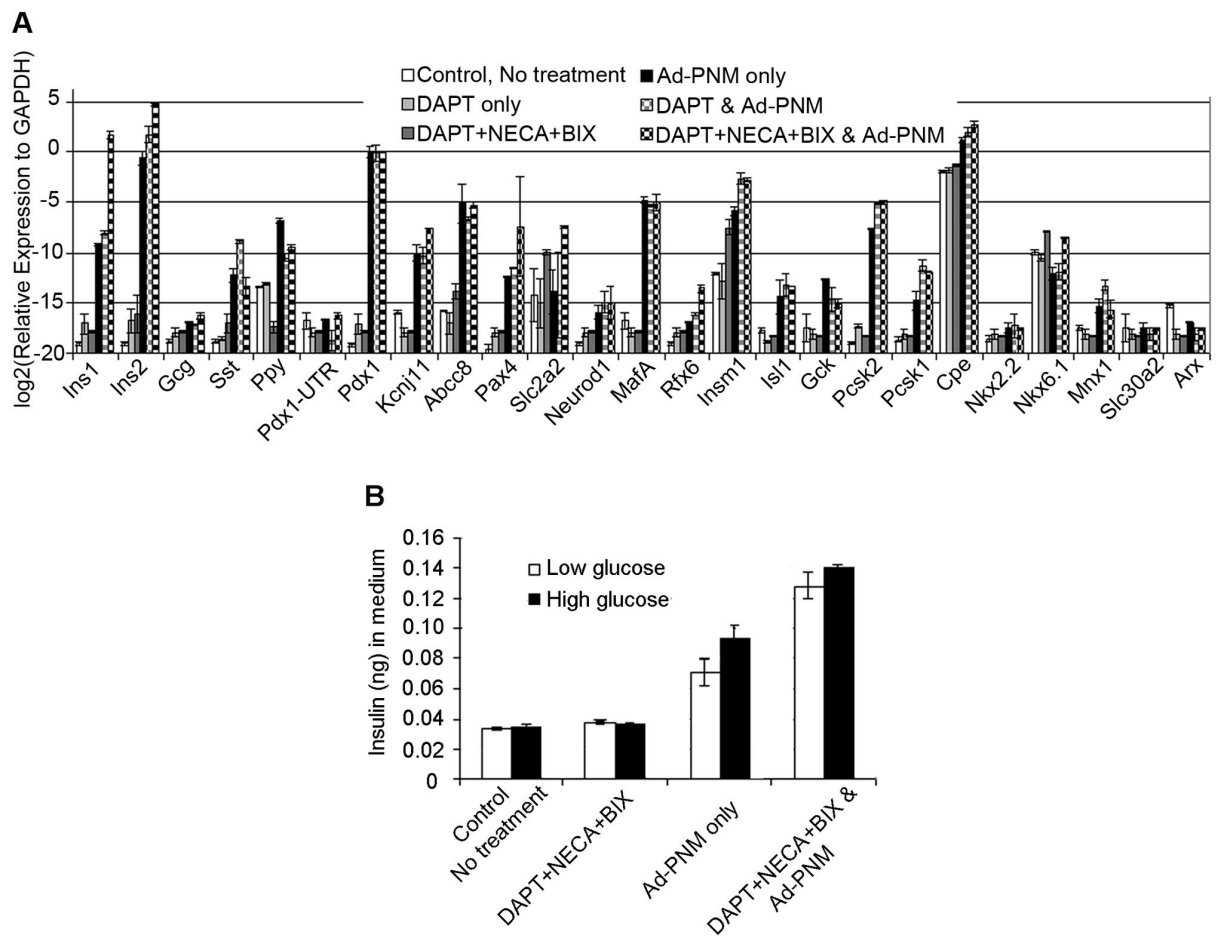
Gene expression in ASH cells provoked by the small molecule combination, with and without *Ad-PNM*. A. qRT-PCR for a panel of beta cell genes. Errors are standard errors, n=3. B. Insulin secretion in low and high glucose. Comparisons are by 1 way ANOVA with Tukey posthoc test and additional t tests.

To extend the analysis we carried out a qRT-PCR analysis using a similar panel of markers to that used for the survey of cell type susceptibility. From these data (Figure **5A**) it can be seen that the level of *Pdx1* and *MafA* expression is the same in all three treatment groups; *Ad-PNM* alone, cells exposed to *Ad-PNM* and DAPT, and cells exposed to *Ad-PNM* and the DNB combination. This suggests that neither DAPT nor the DNB combination affect the viral transduction process or the expression of the viral genes. As we have found above with the ASH cells, *Ad-PNM* altered the gene expression pattern dramatically. Figure **5A** shows that both *insulin* genes were upregulated and the expression level of some important beta cell products also increased, for example the transcription factor genes *Isl1*,*Pax4,Insm1*, the genes encoding proteins for glucose sensing *Kcnj11,Abcc8*, *Gck*, the genes encoding insulin-processing proteins *Cpe*, *Pcsk1&2*. However, not all genes that are known to play an important role in beta cell function were upregulated, for example mRNAs *Nkx2.2* and *6.1* are not elevated. Also worth noting is the lack of endogenous *Pdx1* expression, as this should be present in mature beta cells. Furthermore the levels of the typical beta cell mRNAs, relative to *Gapdh*, are lower than in mouse islet RNA (see Figure S7C of [6]). Immunostaining for several beta cell markers other than insulin (MNX1, RFX6, NEUROD and PAX4 ) gave negative results (data not shown). Overall the results suggest an incomplete type of reprogramming very similar to that seen with AR42j-B13 cells [5]. Moreover, the treatment of ASH cells with DAPT and the DNB combination does not seem to change the qualitative pattern of gene expression. The differences from *Ad-PNM* alone can be accounted for by the increase in the number of responding cells. 

To detect the amount of insulin protein secreted by the transformed cells, and to test for any glucose sensitivity, we measured the amount of insulin in the medium by ELISA after exposure to either low or high glucose (Figure **5B**). A one-way ANOVA followed by Tukey post hoc tests was conducted to examine the effect on insulin secretion of *Ad-PNM*, DNB, and the glucose level in the medium. There was a significant interaction on insulin secretion from the cells: (F(3, 23)=185.3, p<0.001). Insulin secretion was mostly due to the presence of *Ad-PNM* (p<0.001) with a smaller increase associated with DNB (p<0.001). There was an additional small elevation due to the glucose level which just reached statistical significance (t test: p=0.03 for *Ad-PNM* alone and 0.032 for *Ad-*PNM+DNB). The insulin secretion levels were substantially less than the value for mouse islets measured in our lab under the same conditions (2.46ng/µg protein). The insulin content of the cells (AdPNM+DNB) was measured as 105±7.91 pg/µg protein, also less than the level for islets (10.97ng/µg protein). These figures represent the whole cell population, not just the insulin-positive cells. If there are 12% insulin-positive cells in the culture, as shown in Figure **4B**, then the insulin content per cell would be about 0.875ng/µg, still less than the islet level. In addition, although there does appear to be a slight glucose enhancement of insulin secretion, the enhancement from mouse islets is much greater, normally about 5 fold. 

## Discussion

Three days after transduction with *Ad-PNM*, all cell types used in this study showed some degree of *insulin* gene upregulation by RT-PCR. But this in itself may just be due to a direct effect of the input transcription factors. In addition to the *insulin* genes, some other beta cell genes were upregulated in four cell types which were rat AR42J-B13 cells, rat primary hepatocytes, mouse hepatocyte-derived small cells (ASH cells), and rat MAPCs. In these cell types there was also some downregulation of their original major product genes. Immunocytological detection of C-peptide together with insulin protein in the same cells indicated that the insulin is being made by the transformed cells rather than simply being absorbed from the tissue culture medium insulin. This indicates the ability of the *Pdx1*, *Ngn3* and *MafA* combination to lead the transformation of some cell types into a beta cell-like state. However, in no case was the gene expression profile changed to a true beta cell phenotype.

In this study we used eight different cell lines from two different species: rat and mouse. Based on the response of these cell types to *Ad-PNM*, the rat pancreatic exocrine cell line AR42J-B13 cells, rat primary hepatocytes, and mouse ASH cells showed a significant response to *Ad-PNM* by activating many beta cell genes, whereas the three types of fibroblast upregulated few if any apart from the *insulin* genes (Figure **1**). The comparisons cannot be conclusive since the levels of expression of the virus-encoded genes is not exactly the same and the true identity of cell lines is never certain. However we believe that the data are consistent with the idea that competence of cells to give rise to a beta cell-like state is associated with their existing developmental relatedness to beta cells. Pancreatic exocrine and endocrine cells arise from the same progenitor cell population during pancreas development [11] and only diverge at the final stage before terminal cell differentiation. Earlier in embryogenesis the liver and the pancreas arise from neighboring regions of the definitive endoderm. Different signals from surrounding tissues, like FGFs from the cardiac mesoderm and BMP from the septum transversum [12], and activin-β_B_ and FGF2 from the notochord [13] directs the formation of liver and pancreatic buds respectively from definitive endoderm. For this reason pancreatic exocrine cells and hepatocytes may be expected to share some epigenetic memory with beta cells, such that various key genes are located in open chromatin, ready to be turned on in the presence of appropriate transcription factors. Even though these three cell types did not activate all important beta cell genes after transduction with *Ad-PNM*, their higher response is compatible with the idea that developmentally related cells show a higher susceptibility to transformation.

Of thirteen small molecule substances used in this study, the Notch signal blocking agent DAPT gave the best result with a three-fold increase in the number of insulin-positive cells compared to those that were induced with *Ad-PNM* only. This favorable effect of DAPT was probably due to its ability to deplete the Notch signal driven-expression of *Hes1* which suppresses pancreatic endocrine cell formation and which is in most cases reduced by *Ad-PNM* transduction. Our results for DAPT are consistent with the studies performed with developing zebrafish larvae which demonstrated a favorable effect of DAPT on beta cell formation [14,15].

Among chromatin modifying agents tested here, the G9a HMT enzyme inhibitor BIX-01294 was the only one which significantly increased the number of insulin-positive cells. This was probably due to its effect on lowering H3K9Me_2_ level, thereby allowing the active transcription of many genes [16]. The HDAC enzyme inhibitors such as TSA, VPA and SAHA, however, did not increase the number of insulin-positive cells. Their effect was different from that reported by [17] in which HDAC enzyme inhibitors increased the number of endocrine cells in rat embryo pancreatic bud cultured *in vitro*. 

The adenosine agonist NECA is the other small molecule agent that resulted in an almost two-fold increase in the number of insulin-positive mouse hepatocyte-derived small cells. It has recently been showed that NECA acts through the adenosine receptor A2aa and increases the proliferation of beta cells during regeneration in zebrafish[18]. 

When we used these three most effective small molecules (DAPT, BIX and NECA) together on the ASH cells, they increased the number of insulin-positive cells almost six-fold (overall yield~12%). These three agents have different mode of actions and targets in the cell so an approximately additive effect is to be expected.

qRT-PCR and ELISA results demonstrated that there is a small increase in the expression level of many beta cells genes as well as in the amount of the insulin released into the medium from the cells when the DNB combination is used together with *Ad-PNM*. However, the increase in the transcript level as well as the increase of secreted insulin is probably due simply to an increase in the number of the responding cells. This is because there is no upregulation of different beta cell markers in DNB + *Ad-PNM* samples compared to *Ad-PNM* alone. Also, the increase of secreted insulin in high glucose compared to a low glucose environment is very small and much less than real beta cells. We concluded that when used together with *Ad-PNM*, the DNB combination can increase the quantity of transformed cells but not the quality of the transformation 

Our overall conclusions are as follows. Firstly the PNM gene combination has a number of interesting effects but it does not reprogram any of the cell types all the way to genuine beta-cells. Secondly, the partial reprogramming seen may be more pronounced in developmentally related cell types than in unrelated types. Thirdly, the three substance combination DNB gives an enhancement of activity which may prove useful for this type of reprogramming in future.
